# Supplementary figures and images for: Inhibition of PHB1/PHB2 suppresses atherosclerotic plaque formation by interrupting PI3K/AKT/mTOR signaling
Source: PLoS One. 2025 Apr 1;20(4):e0320509. doi: 10.1371/journal.pone.0320509 (PMC11960914; doi:10.1371/journal.pone.0320509)

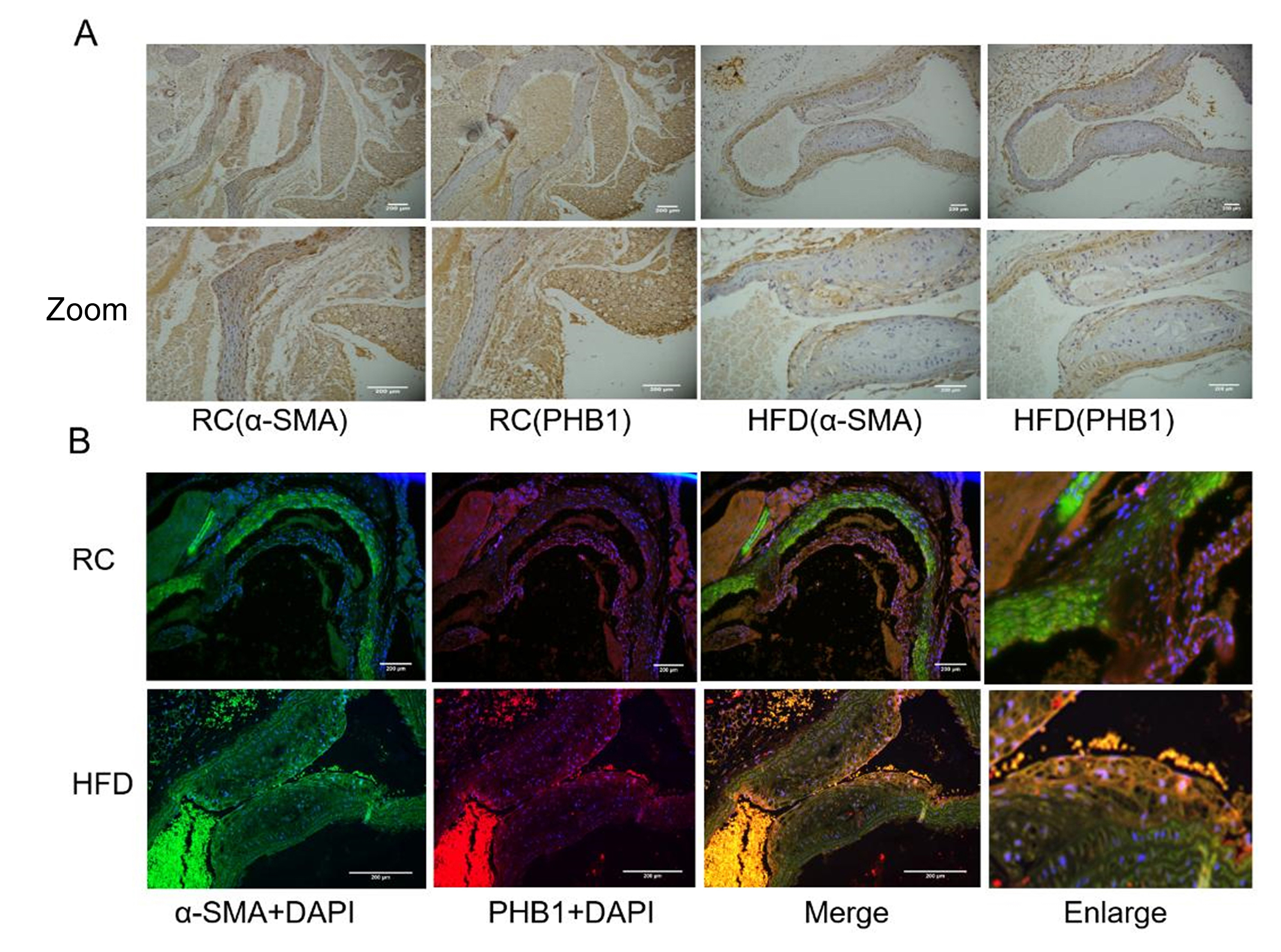

Supplement: S1 Fig — (A) Representative immunohistochemistry images of α-SMA (a marker of VSMCs) and PHB1 in ApoE−/− mice fed an HFD and regular chow (RC), Brown color represents positive cells; (B) Representative immunofluorescence images of α-SMA and PHB1 in ApoE−/− mice with HFD and RC; scale bars: 200 μm. (TIF) [file pone.0320509.s001.tif]

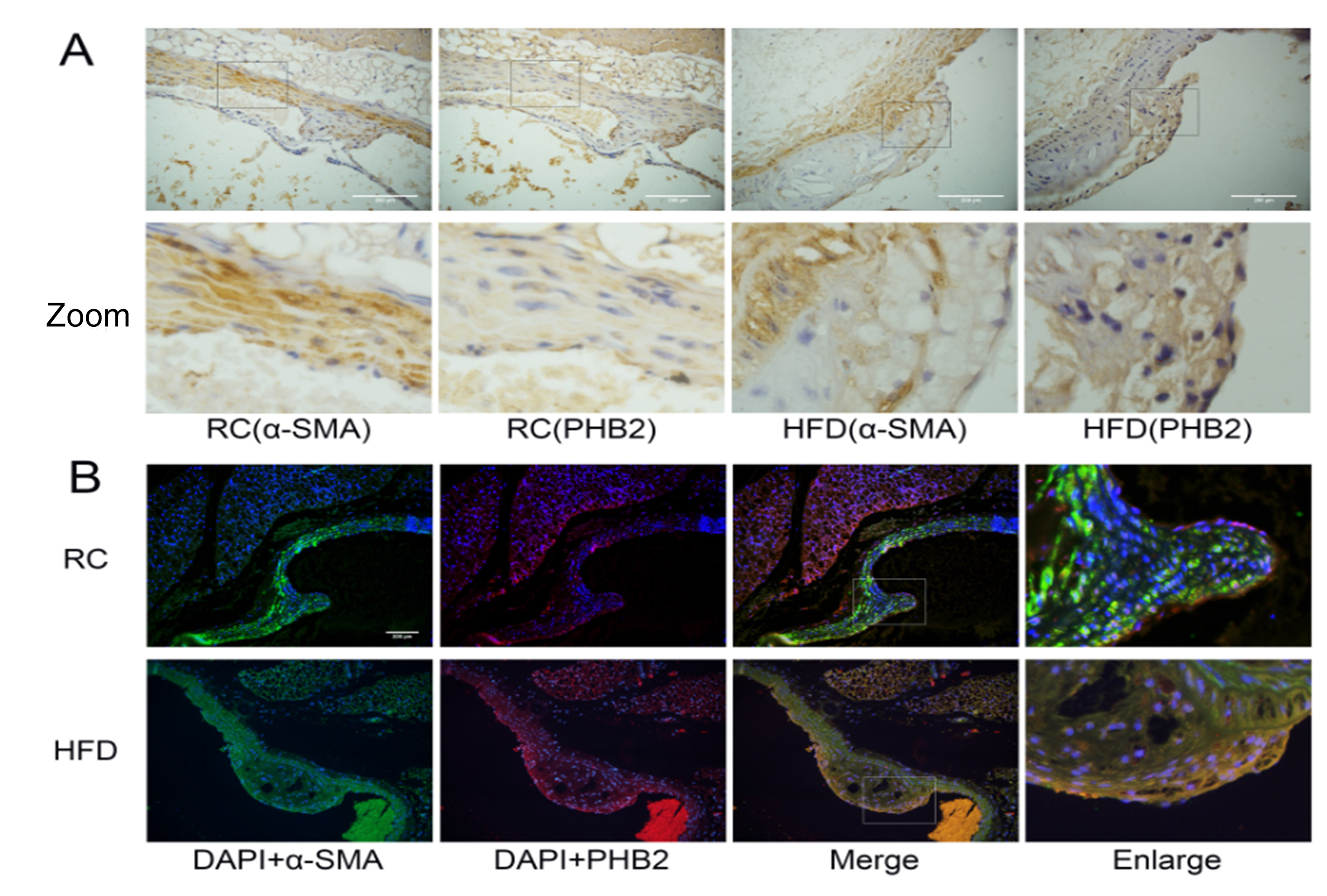

Supplement: S2 Fig — (A) Representative immunohistochemistry images of α-SMA (a marker of VSMCs) and PHB2 in ApoE−/− mice with HFD and RC; (B) Representative immunofluorescence images of α-SMA and PHB2 in ApoE−/− mice with HFD and RC; scale bars: 200 μm. (TIF) [file pone.0320509.s002.tif]

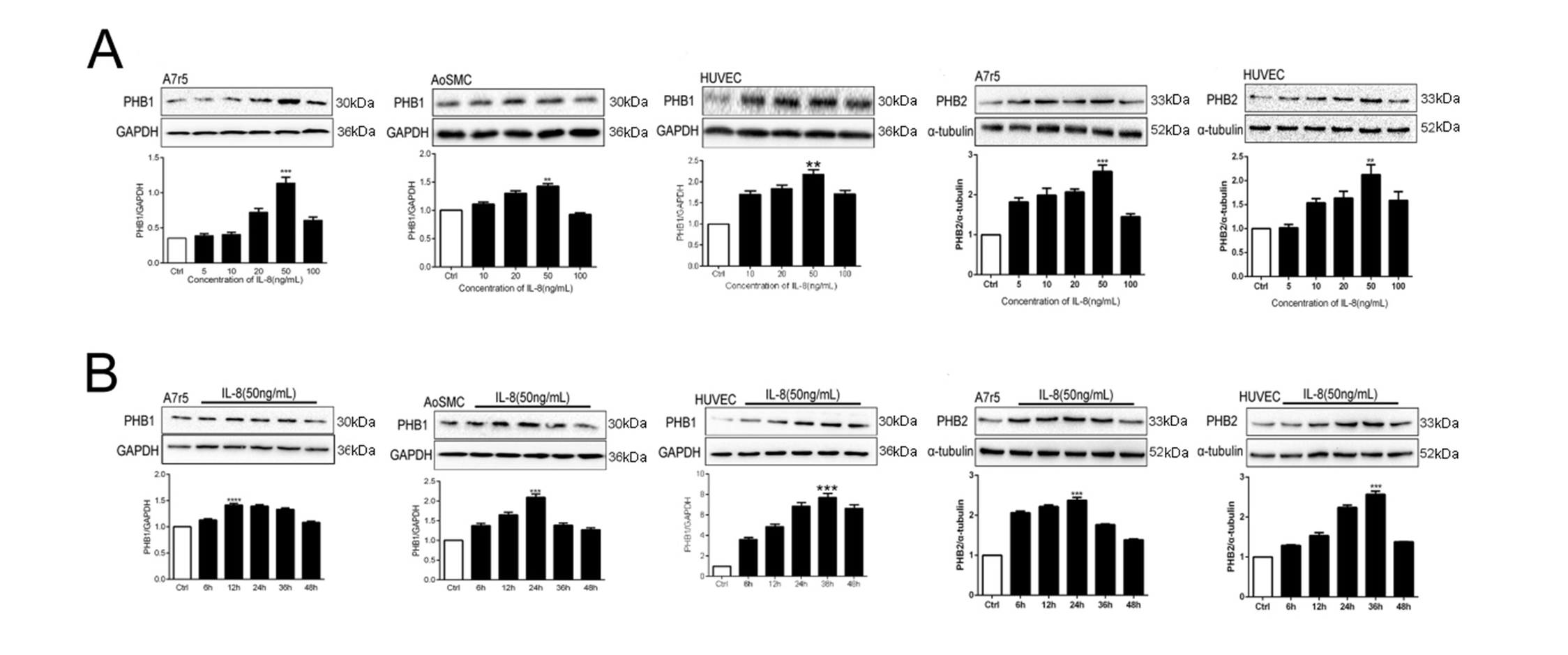

Supplement: S3 Fig — (A) Western blotting showing the expression of PHB1 and PHB2 in HUVEC, A7r5, and AosMC cells treated with IL-8 (0–100 ng/mL). (B) Western blotting showing the expression of PHB1 and PHB2 in HUVEC, A7r5, and AosMC cells treated with IL-8 (50 ng/L) (0–48 h). All data are expressed as the mean ± SD (n = 5). * p < 0.05, **p < 0.01, ***p < 0.001. (TIF) [file pone.0320509.s003.tif]

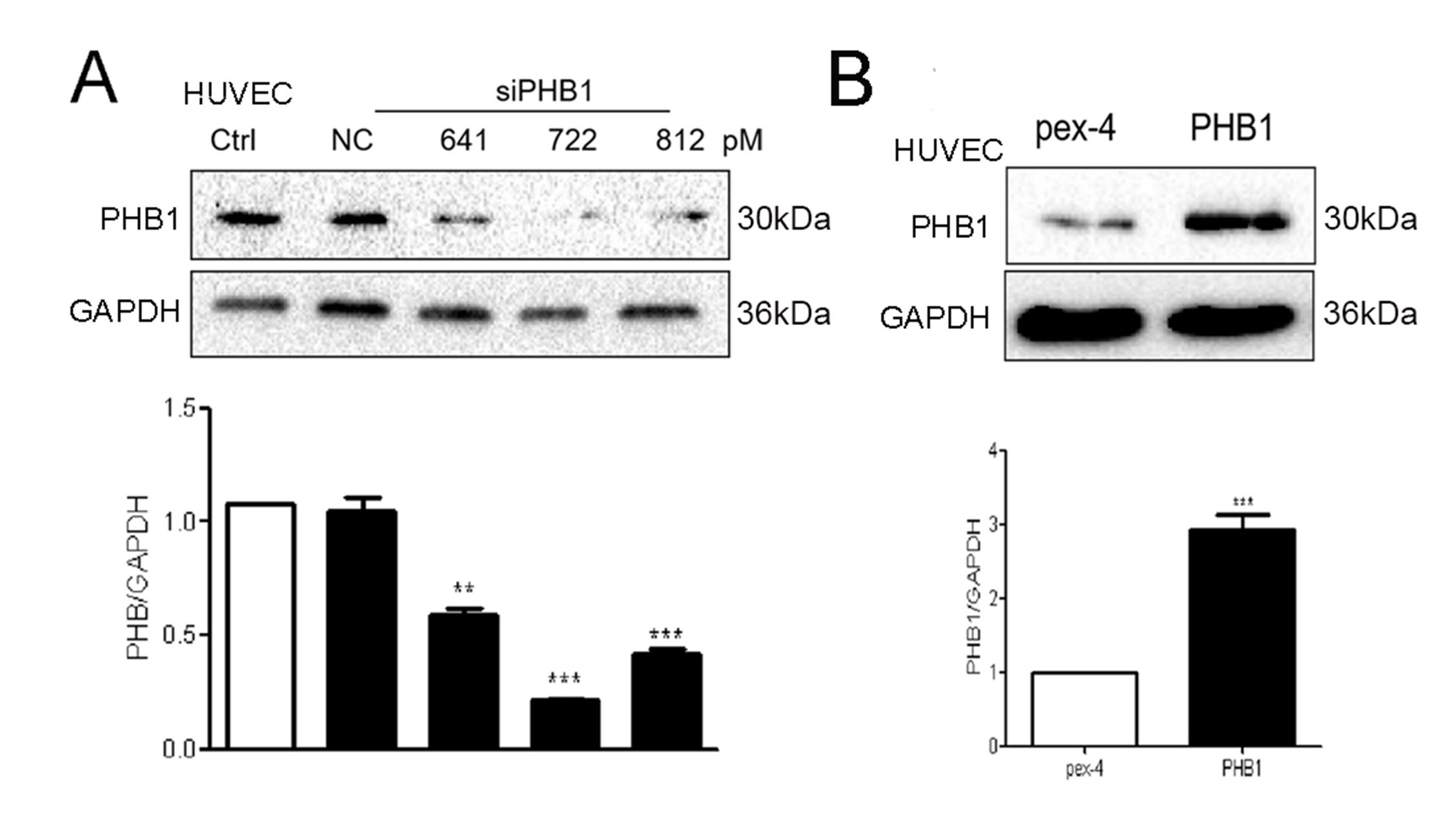

Supplement: S4 Fig — (A) Western blotting confirmed the efficiency of siPHB1 in HUVECs; siPHB1 represents siRNA-PHB1. NC represents negative siRNA used as control. (B) Western blotting confirmed the efficiency of the pex-4-PHB1 plasmid in HUVECs. Pex-4 represents the empty vector, whereas PHB1 represents the pex-4-PHB1 plasmid. All data are expressed as the mean ± SD (n = 5). * p < 0.05, **p < 0.01, ***p < 0.001. (TIF) [file pone.0320509.s004.tif]

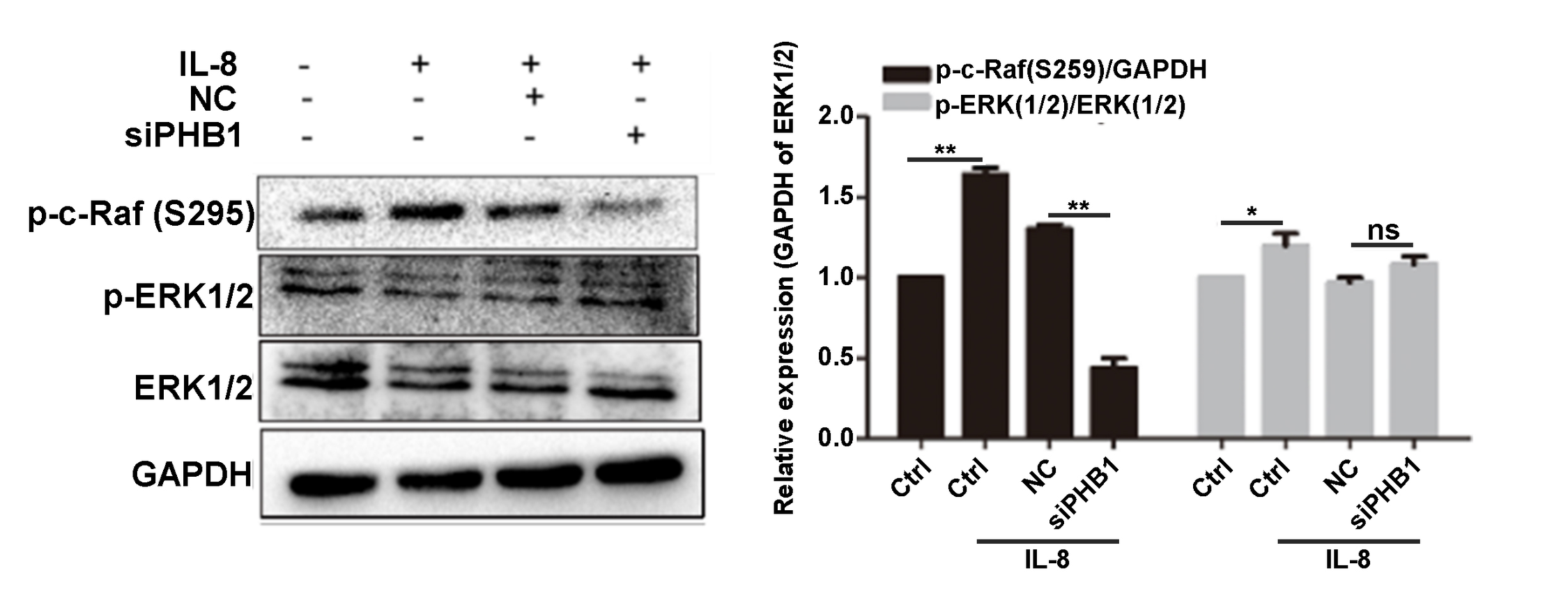

Supplement: S5 Fig — Western blotting showing the expression of p-c-Raf (S295), p-ERK1/2 and ERK1/2. All data are expressed as the mean ± SD (n = 5). * p < 0.05, **p < 0.01, ***p < 0.001. (TIF) [file pone.0320509.s005.tif]
