# Supplementary material for: Inhibition of PHB1/PHB2 suppresses atherosclerotic plaque formation by interrupting PI3K/AKT/mTOR signaling
Source: PLoS One. 2025 Apr 1;20(4):e0320509. doi: 10.1371/journal.pone.0320509 (PMC11960914; doi:10.1371/journal.pone.0320509)
Supplement: Attachment — Submitted filename: Dear.doc [file pone.0320509.s007.doc]

Dear editors,

Thanks for your suggestions. We have uploaded the completed Human Participants Research Checklist as file type “Other”, and also uploaded the original approval document that received from my ethics committee. The month and year of the start and end of the recruitment period for this study was added in the manuscript.

We look forward to hearing from you soon.

Yours sincerely,

Ying Gao

Corresponding author

E-mail: [gaoying200018@126.com](mailto:gaoying200018@126.com)
